# Large Language Models Take on Cardiothoracic Surgery: A Comparative Analysis of the Performance of Four Models on American Board of Thoracic Surgery Exam Questions in 2023

**DOI:** 10.7759/cureus.65083

**Published:** 2024-07-22

**Authors:** Zain Khalpey, Ujjawal Kumar, Nicholas King, Alyssa Abraham, Amina H Khalpey

**Affiliations:** 1 Khalpey AI Lab, Department of Cardiothoracic Surgery, HonorHealth, Scottsdale, USA; 2 Department of Research, Applied & Translational AI Research Institute (ATARI), Scottsdale, USA; 3 School of Clinical Medicine, University of Cambridge, Cambridge, GBR; 4 Department of Research, DataKinetic, Austin, USA; 5 Department of Research, GlobalHealth AI LLC, Scottsdale, USA

**Keywords:** claude 2, med-palm 2, chatgpt, sesats, american board of thoracic surgery, cardiothoracic surgical education, board exams, large language models, artificial intelligence

## Abstract

Objectives

Large language models (LLMs), for example, ChatGPT, have performed exceptionally well in various fields. Of note, their success in answering postgraduate medical examination questions has been previously reported, indicating their possible utility in surgical education and training. This study evaluated the performance of four different LLMs on the American Board of Thoracic Surgery’s (ABTS) Self-Education and Self-Assessment in Thoracic Surgery (SESATS) XIII question bank to investigate the potential applications of these LLMs in the education and training of future surgeons.

Methods

The dataset in this study comprised 400 best-of-four questions from the SESATS XIII exam. This included 220 adult cardiac surgery questions, 140 general thoracic surgery questions, 20 congenital cardiac surgery questions, and 20 cardiothoracic critical care questions. The GPT-3.5 (OpenAI, San Francisco, CA) and GPT-4 (OpenAI) models were evaluated, as well as Med-PaLM 2 (Google Inc., Mountain View, CA) and Claude 2 (Anthropic Inc., San Francisco, CA), and their respective performances were compared. The subspecialties included were adult cardiac, general thoracic, congenital cardiac, and critical care. Questions requiring visual information, such as clinical images or radiology, were excluded.

Results

GPT-4 demonstrated a significant improvement over GPT-3.5 overall (87.0% vs. 51.8% of questions answered correctly, p < 0.0001). GPT-4 also exhibited consistently improved performance across all subspecialties, with accuracy rates ranging from 70.0% to 90.0%, compared to 35.0% to 60.0% for GPT-3.5. When using the GPT-4 model, ChatGPT performed significantly better on the adult cardiac and general thoracic subspecialties (p < 0.0001).

Conclusions

Large language models, such as ChatGPT with the GPT-4 model, demonstrate impressive skill in understanding complex cardiothoracic surgical clinical information, achieving an overall accuracy rate of nearly 90.0% on the SESATS question bank. Our study shows significant improvement between successive GPT iterations. As LLM technology continues to evolve, its potential use in surgical education, training, and continuous medical education is anticipated to enhance patient outcomes and safety in the future.

## Introduction

Large language models (LLMs) such as ChatGPT, released by OpenAI (San Francisco, CA) [1], have been shown to perform exceptionally well across various fields such as medicine, law, and management. The performance of LLMs, specifically ChatGPT, on medical licensing and general surgical board exam questions has been reported previously [2-4], implying the significant potential for their use in surgical education and training. What is not reported, however, is the performance of LLMs on specialist topics, such as those from a cardiothoracic surgical board exam question bank. These require knowledge of and the ability to process much more specialized, niche information that will be less well represented in training datasets than knowledge to answer medical licensing or general surgical board exam questions.

Cardiothoracic surgery is a highly specialized field, and cardiothoracic surgeons must have extensive knowledge and skill in the surgical treatment of conditions involving the heart, lungs, esophagus, and other intrathoracic organs. Self-Education and Self-Assessment in Thoracic Surgery (SESATS) is a sophisticated online educational and assessment platform designed specifically for cardiothoracic surgeons. It offers a comprehensive, self-directed curriculum covering the entire range of knowledge required for cardiothoracic surgical practice. Successful completion of SESATS is essential for surgeons to maintain their professional knowledge, enhance their skills, and uphold board certification standards. Additionally, it serves as a valuable study tool for those preparing for the American Board of Thoracic Surgery (ABTS) examinations.

This study focuses on evaluating and comparing the performance of various large language models on the SESATS XIII board questions from the ABTS. This study evaluated ChatGPT (GPT-3.5 and GPT-4 models from OpenAI) as well as Med-PaLM 2 (Google Inc., Mountain View, CA) and Claude2 (Anthropic Inc., San Francisco, CA). We also aim to explore the implications of LLM technology for surgical education and training in the field of cardiothoracic surgery.

## Materials and methods

Dataset for model testing

The dataset used in this study for the evaluation of the four LLMs consisted of 400 questions, which were obtained from the SESATS XIII question bank from 2023 [5]. Questions covered topics including disorders of the lung, chest wall, and mediastinum, acquired and congenital heart disease, and issues in critical care. These questions were categorized into four main categories: adult cardiac surgery (n = 220, 55.0%), general thoracic surgery (n = 140, 35.0%), congenital cardiac surgery (n = 20, 5.0%), and critical care (n = 20, 5.0%). Any questions requiring visual inputs (clinical images or radiology) were excluded from the dataset, as not all LLMs being evaluated in this study could interpret such information.

Large language models and performance evaluation

In this study, we primarily utilized the ChatGPT language model developed by OpenAI to evaluate its performance on the dataset of questions. We initially tested both the GPT-3.5 and GPT-4 models. To evaluate the model's performance, we manually entered the questions into the relevant website for each LLM and compared the answers provided by the model to those of the official SESATS answer key. To ensure consistency and avoid potential biases, each question was individually copy-pasted into a new session of the relevant LLM, with the session being reset after each question. Questions were presented to the models in the same order as they appear in the SESATS XIII question bank. No preprocessing of the questions was performed beyond removing any visual elements not compatible with the text-based interface of the LLMs.

Two independent board-certified cardiothoracic surgeons verified the answers and resolved any discrepancies through discussion. Additional investigations were undertaken using Med-PaLM 2 [6] and Claude 2 [7]. Large language models and comparisons were undertaken between all four groups. Since this work was conducted, newer generations of LLM have subsequently been released, such as Claude 3 and GPT-4o. Future work from our group will evaluate these models to compare them to the models evaluated in this study.

Statistical analysis

The performance of both the GPT-3.5 and GPT-4 models was evaluated by calculating the overall accuracy, defined as the proportion of correct answers relative to the total number of questions. Subgroup analyses were conducted to assess the models' performance across various categories of cardiothoracic surgery. Chi-square statistical tests were used to compare the performances of GPT-3.5 and GPT-4, with p-values less than 0.05 deemed to be statistically significant. For each category and overall performance, 2x2 contingency tables were constructed, comparing the number of correct and incorrect answers between the two models. All analyses were performed using GraphPad Prism v10.2.0 for macOS (GraphPad Software Inc., Boston, MA) [8], using the software's built-in chi-square test function with default settings.

Ethical considerations

This study did not involve human subjects or patient data, focusing solely on publicly available educational materials. Nevertheless, we adhered to ethical guidelines in AI research, ensuring responsible use of the LLMs and maintaining the integrity of the SESATS question bank. All LLMs were accessed through their official, publicly available interfaces, and no attempts were made to modify or manipulate their underlying algorithms or training data.

## Results

A full comparison of the accuracy scores of the four LLMs is shown below in Table 1.

**Table 1:** Full comparison of the accuracy scores of the four large language models (LLMs). There were significant differences between the overall performances of the four LLMs, as well as in the adult cardiac and general thoracic subspecialties. The data represented here are the number and percentage of questions in each category answered correctly by the LLM, presented as N (%), with a significance threshold of 0.05.

Category	GPT-3.5	Med-PaLM 2	Claude 2	GPT-4	p-value
Overall	207 (51.8%)	223 (55.8%)	209 (52.3%)	348 (87.0%)	< 0.0001
Adult cardiac surgery	108 (49.1%)	116 (52.7%)	108 (49.1%)	192 (87.3%)	< 0.0001
General thoracic surgery	80 (57.1%)	86 (61.4%)	81 (57.9%)	126 (90.0%)	< 0.0001
Congenital cardiac surgery	7 (35.0%)	9 (45.0%)	8 (40.0%)	14 (70.0%)	0.1210
Critical care	12 (60.0%)	12 (60.0%)	12 (60.0%)	16 (80.0%)	0.4510

Overall performance

**Figure 1:**
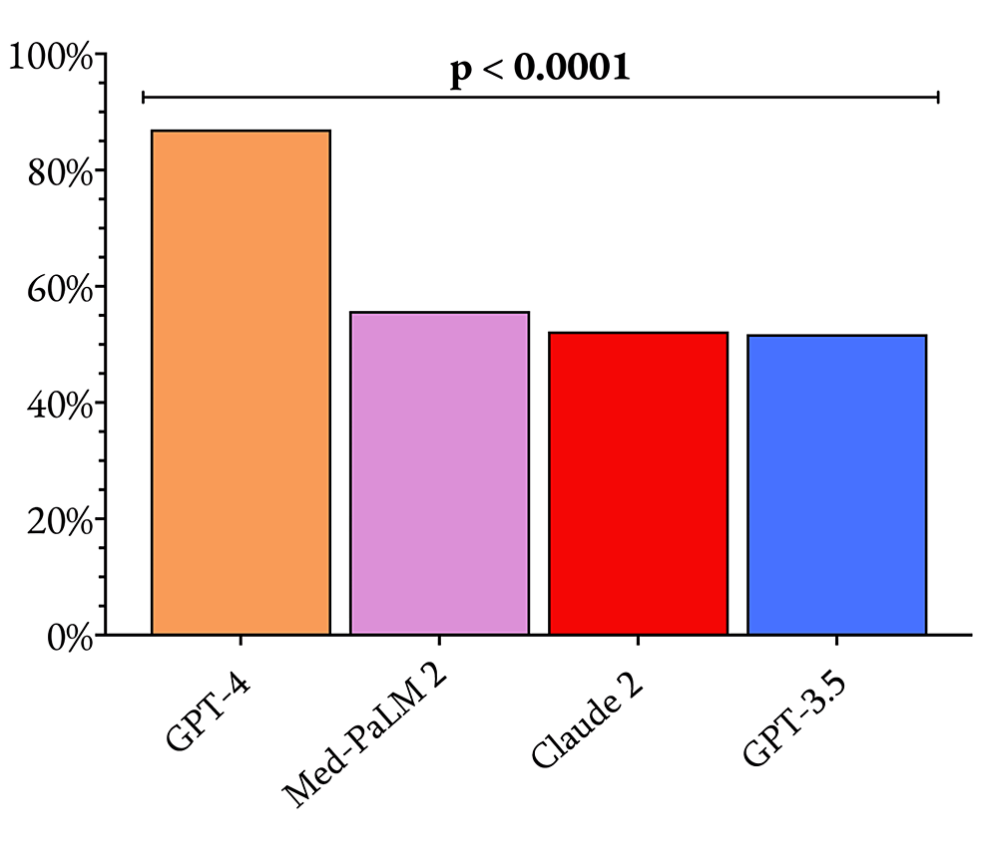
Comparison of the overall performance of the four large language models evaluated in this study The data plotted are the proportion of questions that each model answered correctly, with a p-value of 0.05 considered the significance threshold.

The GPT-4 model exhibited a remarkable improvement over its predecessor, GPT-3.5, in terms of overall accuracy on the SESATS question set. GPT-4 correctly answered 87.0% (348 out of 400) of the total questions, while GPT-3.5 managed to answer only 51.8% (207 out of 400) correctly. This difference was statistically significant (p < 0.0001), underscoring the substantial advancements made in the GPT-4 model. The other two models evaluated, Med-PaLM 2 and Claude 2, exhibited performance levels more akin to GPT-3.5, with overall accuracies of 55.8% and 52.3% (209 and 223 out of 400), respectively. The overall performances of the four LLMs are shown in Figure 1.

Performance by subspecialty

**Figure 2:**
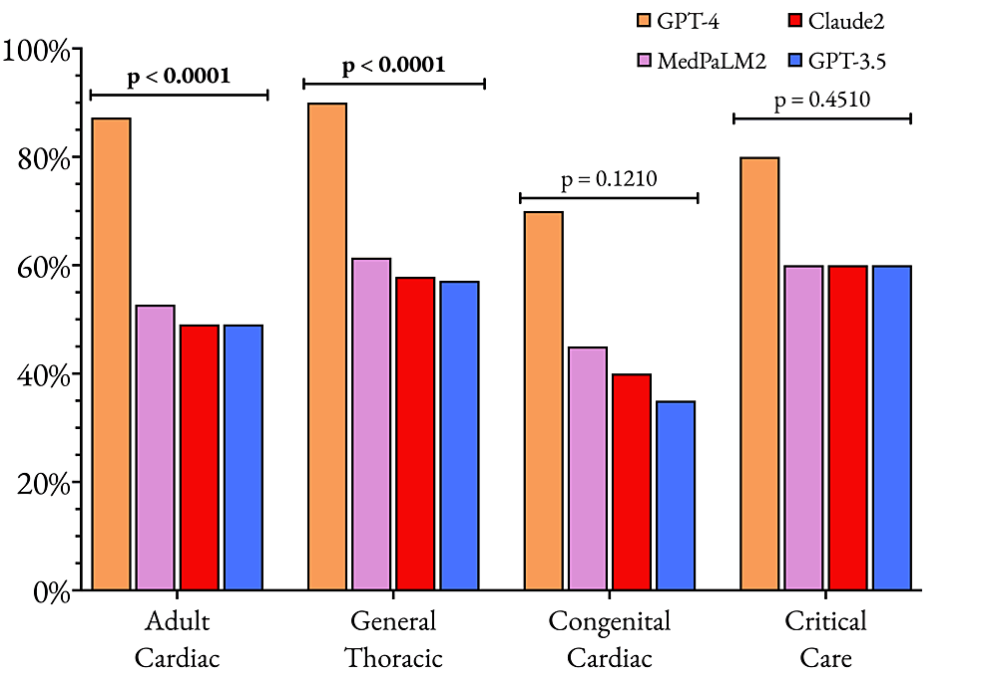
Comparison of the performances of the four large language models across the four subspecialties represented within the SESATS XIII question bank The data plotted are the proportion of questions that each model answered correctly, with a p-value of 0.05 considered the significance threshold. SESATS: Self-Education and Self-Assessment in Thoracic Surgery

Further analysis of the different models' performance across various cardiothoracic surgery subspecialties revealed that GPT-4 consistently outperformed the other models, showcasing its versatility and adaptability, as shown in Figure 2. In the adult cardiac surgery subset, which comprised the largest number of questions (n = 220), GPT-4 achieved an impressive accuracy of 87.3%, significantly surpassing GPT-3.5's accuracy of 49.1% (192 vs. 108 correct, p < 0.0001). Similarly, in the general thoracic surgery subset (n = 140), GPT-4 attained an accuracy of 90.0%, markedly higher than GPT-3.5's 57.1% (126 vs. 80 correct, p < 0.0001). These results highlight GPT-4's superior ability to comprehend and navigate complex clinical scenarios in the two most prominent subspecialties of cardiothoracic surgery.

Although the question sets for congenital cardiac surgery (n = 20) and critical care (n = 20) were notably smaller, GPT-4 still demonstrated better performance compared to GPT-3.5. In congenital cardiac surgery, GPT-4 achieved an accuracy of 70.0%, compared to GPT-3.5's 35.0% (14 vs. 7 correct, p = 0.1210). For critical care questions, GPT-4 attained an accuracy of 80.0%, while GPT-3.5 managed 60.0% (16 vs. 12 correct, p = 0.4510). Despite the smaller sample sizes in these subspecialties, the results suggest that GPT-4's advanced language processing capabilities extend to these specialized areas of cardiothoracic surgery.

Med-PaLM 2 and Claude 2 displayed relatively consistent performance across all subspecialties, with accuracy rates ranging from 45%-61% for Med-PaLM 2 and 40%-60% for Claude 2. Their performance profiles more closely resembled those of GPT-3.5 than GPT-4, indicating that the advancements made in GPT-4 have not been paralleled in these other state-of-the-art language models.

Iterative improvement from GPT-3.5 to GPT-4

**Figure 3:**
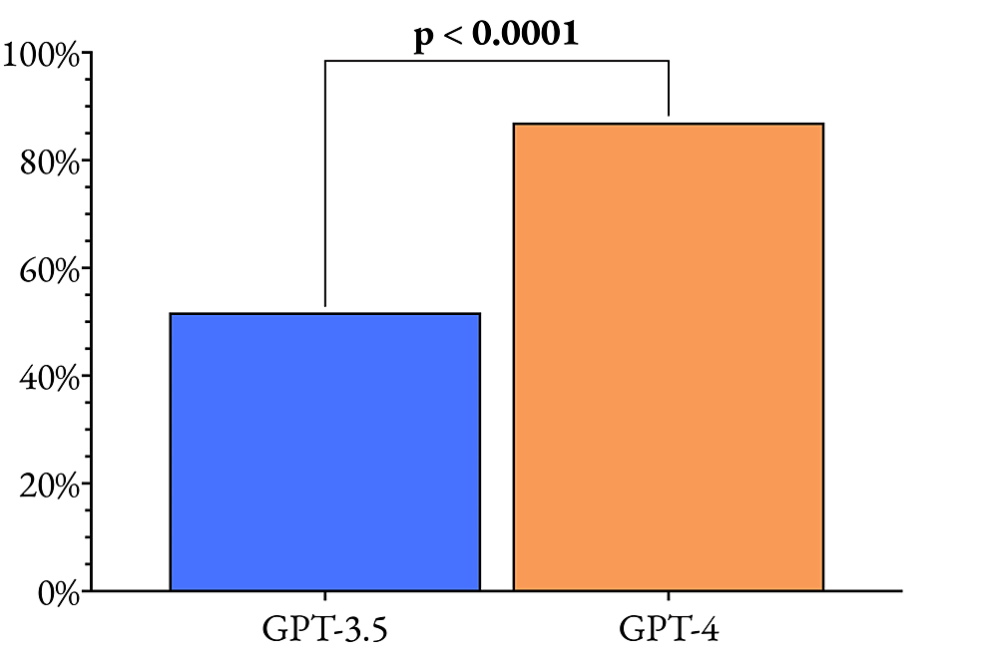
Comparison of the overall performance of GPT-3.5 and GPT-4 to evaluate the iterative improvement between these two models. The data plotted are the proportion of questions that each model answered correctly, with a p-value of 0.05 considered the significance threshold.

One of the most striking findings of this study is the substantial improvement in performance between the GPT-3.5 and GPT-4 models, highlighting the rapid advancements in natural language processing (NLP) and machine learning techniques. The overall accuracy of GPT-4 (87.0%) was significantly higher than that of GPT-3.5 (51.8%), with a p-value < 0.0001, demonstrating the effectiveness of the iterative refinements and optimizations implemented in the newer model. The iterative improvement in overall performance is highlighted in Figure 3, with a full comparison in Table 2.

**Table 2:** Full comparison of accuracy scores of the GPT-3.5 and GPT-4 large language models (LLMs). There was a significant difference between the overall performances of GPT-3.5 and GPT-4, as well as in the adult cardiac and general thoracic subspecialties. The data represented here are the number and percentage of questions in each category answered correctly by the LLM, presented as N (%), with a significance threshold of 0.05.

Category	GPT-3.5	GPT-4	p-value
Overall	207 (51.8%)	348 (87.0%)	< 0.0001
Adult cardiac surgery	108 (49.1%)	192 (87.3%)	< 0.0001
General thoracic surgery	80 (57.1%)	126 (90.0%)	< 0.0001
Congenital cardiac surgery	7 (35.0%)	14 (70.0%)	0.0562
Critical care	12 (60.0%)	16 (80.0%)	0.3008

**Figure 4:**
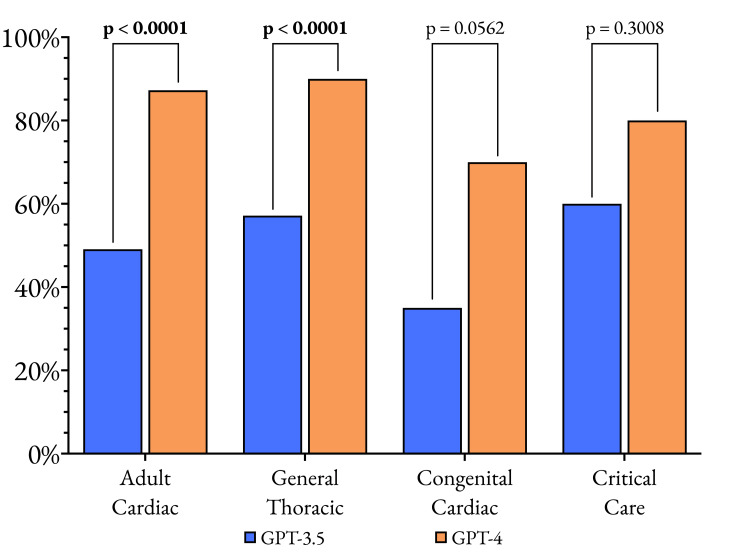
Comparison of the performance of GPT-3.5 and GPT-4 across the four subspecialties in the SESATS XIII question bank to evaluate the iterative improvement between these two models. The data plotted are the proportion of questions that each model answered correctly, with a p-value of 0.05 considered the significance threshold. SESATS: Self-Education and Self-Assessment in Thoracic Surgery

This marked improvement was consistently observed across all subspecialties of cardiothoracic surgery (Figure 4), suggesting that the enhancements in GPT-4 are not limited to specific domains but represent a comprehensive upgrade in the model's ability to understand, process, and generate relevant information. In the adult cardiac surgery subset, GPT-4's accuracy of 87.3% was a dramatic improvement over GPT-3.5's 49.1% (p < 0.0001). Similarly, in general thoracic surgery, GPT-4 achieved an impressive 90.0% accuracy compared to GPT-3.5's 57.1% (p < 0.0001).

Even in the smaller subsets of congenital cardiac surgery and critical care, where the differences were less pronounced due to the limited number of questions, GPT-4 still outperformed GPT-3.5. In congenital cardiac surgery, GPT-4 achieved a 70.0% accuracy compared to GPT-3.5's 35.0% (p = 0.0562), while in critical care, GPT-4 attained an 80.0% accuracy versus GPT-3.5's 60.0% (p = 0.3008).

The iterative improvement observed between GPT-3.5 and GPT-4 can be attributed to several factors, including advancements in model architecture, training data, and fine-tuning techniques. The GPT-4 model likely benefits from a larger and more diverse training dataset, allowing it to better capture the nuances and complexities of medical language and clinical scenarios. Additionally, improvements in the model's attention mechanisms and context understanding may contribute to its enhanced performance in processing lengthy and intricate questions, such as those found in the SESATS question set.

Furthermore, the superior performance of GPT-4 compared to other state-of-the-art language models, such as Med-PaLM 2 and Claude 2, suggests that the iterative refinements implemented in the GPT series have been particularly effective in the domain of medical language processing. This observation underscores the potential for the GPT architecture to serve as a foundation for future advancements in AI-assisted clinical decision support and surgical education.

In conclusion, the iterative advancements demonstrated by GPT-4 over its predecessor, GPT-3.5, across all cardiothoracic surgical subspecialties underscore the rapid progress in NLP models. This study highlights the transformative potential of evolving NLP technologies in various facets of medical practice, such as clinical decision-making, surgical training, and knowledge assessment. The compelling evidence presented supports the continued refinement and application of language models like GPT-4 to meet the dynamic needs of the medical community.

The findings of this study particularly emphasize the significant strides made by the GPT-4 model in comprehending and processing intricate clinical information across diverse cardiothoracic surgical subspecialties. GPT-4's performance was notably superior in larger question sets related to adult cardiac and general thoracic surgery, markedly surpassing GPT-3.5 and other tested models. Although GPT-4 also exhibited improved performance in the smaller subsets of congenital cardiac surgery and critical care, the performance differences were less pronounced, likely due to the limited number of questions in these categories. Overall, these results highlight the remarkable potential of GPT-4 and similar advanced language models to revolutionize clinical decision support, surgical education, and knowledge assessment within the realm of cardiothoracic surgery.

## Discussion

The advent of advanced artificial intelligence (AI) tools such as ChatGPT has generated both excitement and concern within the medical community, particularly in the field of surgery. The integration of these tools holds significant promise for revolutionizing surgical education and training, specifically within the field of cardiothoracic surgery, which is complex and ever-changing. The results of this study demonstrate the remarkable potential of advanced AI models, such as ChatGPT, particularly the GPT-4 model, in revolutionizing surgical education and training. GPT-4's impressive accuracy rate of 87.0% on the SESATS board questions consistently outperformed other large language models, including GPT-3.5, Med-PaLM 2, and Claude 2, across all subspecialties of cardiothoracic surgery. This performance underscores GPT-4's ability to comprehend and process complex clinical information, marking a significant step forward from previous iterations.

As the field of cardiothoracic surgery continues to evolve rapidly, practitioners must maintain their knowledge and skills to ensure optimal patient outcomes. The integration of LLMs like GPT-4 into surgical education could provide an interactive and adaptive learning platform tailored to individual learners. Moreover, LLMs may serve as a valuable resource for continuous medical education (CME), enabling surgeons to stay abreast of the latest advancements in their field [9]. By reducing the number of errors made by surgeons and improving the quality of surgical education, AI models like ChatGPT have the potential to significantly enhance patient care and outcomes.

Strengths and limitations

This study has several notable strengths. Firstly, the use of the specialized SESATS XIII question bank provides a robust and relevant dataset for evaluating LLM performance in cardiothoracic surgery. The verification of answers by board-certified cardiothoracic surgeons ensures the reliability and clinical relevance of our assessments. Additionally, our study demonstrates the significant improvement from GPT-3.5 to GPT-4, highlighting the rapid advancements in natural language processing and its potential applications in surgical education.

However, we acknowledge several limitations of our study. The dataset, while specialized, is relatively small at 400 questions, which may not fully capture the breadth and depth of knowledge required in cardiothoracic surgery. The exclusion of questions requiring visual information limits the real-world applicability of our findings. While this was necessary to compare a wider range of LLMs, as not all could interpret visual information, given the critical role of visual interpretation in surgical decision-making, this exclusion may limit the generalizability of the findings to real-world clinical scenarios.

Another limitation is the study's focus on multiple-choice questions. While this format is commonly used in board certification exams, it does not assess other crucial aspects of surgical competence, such as practical skills, clinical judgment, and decision-making in complex, real-time situations. The performance of AI models on multiple-choice questions may not directly translate to their potential utility in the operating room or other aspects of surgical education and practice. Furthermore, the study did not compare the AI models' performance to that of human surgeons or trainees at different levels of expertise. Without this benchmark, one cannot fully contextualize the significance of the AI models' accuracy rates and determine whether they truly achieve or exceed human levels of performance.

We also recognize that the field of AI is rapidly evolving, with new models and techniques emerging at an unprecedented pace. The current study evaluated the performance of GPT-3.5, GPT-4, Med-PaLM 2, and Claude 2 which were considered state-of-the-art at the time of the research. However, subsequent, more advanced models may soon surpass these results, potentially limiting the long-term relevance of the findings. Lastly, we recognize that the AI models' responses are based on their training data, which may contain biases, gaps, or inaccuracies. This could lead to biased or incomplete responses in certain areas, particularly in the context of the highly specialized and constantly evolving field of cardiothoracic surgery. Future studies should address these limitations by incorporating a larger, more diverse question set, including visual elements, and comparing AI performance with that of human surgeons at various levels of training.

Benefits of LLMs for surgeons

Large language models offer several benefits to surgical practice. It can enhance decision-making by providing real-time data analysis and evidence-based recommendations, leading to more accurate and informed decisions during surgical procedures [10,11]. The model's adaptability to individual learning needs and preferences can result in personalized surgical training, ultimately improving patient outcomes [12]. ChatGPT also enables rapid access to the latest medical research and guidelines, ensuring that surgeons are up-to-date with current best practices and reducing the likelihood of errors [13]. Furthermore, it can facilitate interdisciplinary collaboration by streamlining communication between surgical teams and other medical professionals, optimizing patient care [14]. By providing instant access to relevant information and accurately analyzing large volumes of data, ChatGPT can reduce the cognitive load on surgeons, allowing them to focus on critical aspects of surgery and improving their performance [15,16].

Concerns about surgeons’ use of LLMs

However, the advent of advanced AI models in medicine has also generated concerns within the medical community. The potential for incorrect or misleading information generated by these models necessitates ongoing evaluation and refinement as they are implemented in educational and clinical settings [17,18]. Additionally, the controversial aspect of AI's role in medicine has led to heated debates regarding its future implications, such as the potential displacement of human expertise and the ethical considerations surrounding AI-assisted decision-making. Issues such as the privacy of personal information, transparency, and cybersecurity are significant concerns [19]. Furthermore, the potential for bias in the training of AI systems is a crucial consideration [20].

Other potential concerns include new surgeons' overreliance on AI, which may hinder the development of their clinical judgment and decision-making skills. The use of AI in medical decision-making also raises several ethical concerns, including questions surrounding accountability, patient autonomy, and informed consent. Ensuring the privacy and security of patient information is paramount when using AI systems like ChatGPT, and the potential for data breaches could lead to significant consequences. Misinterpretation or misunderstanding of ChatGPT's recommendations by surgeons may lead to errors in clinical practice. Moreover, ChatGPT models are based on the data they have been trained on, which could potentially introduce biases that may affect the quality of recommendations provided to surgeons.

Despite these concerns, it is essential to recognize that GPT-4 algorithms are designed to enhance, not replace, the skills of surgeons. By integrating AI technologies such as ChatGPT into surgical training and practice, we can leverage the strengths of both human expertise and AI capabilities to improve patient outcomes and advance the field of surgery.

In conclusion, while the integration of advanced AI models like ChatGPT in surgical education and training holds immense promise, it is essential to approach their implementation with caution and to engage in open discussions about their potential benefits and drawbacks. As we navigate this new frontier in medical education and practice, collaboration between AI developers, medical professionals, and ethicists will be crucial in ensuring that these technologies are harnessed in a manner that prioritizes patient safety and well-being. With successive iterations of AI models continually improving, the future holds great potential for even more advanced tools to enhance surgical practice and education.

## Conclusions

ChatGPT, particularly when using the GPT-4 iteration, exhibited a significant capability in comprehending intricate cardiothoracic surgical clinical information, achieving an overall accuracy rate of 87.0% on the SESATS questions. This performance exceeds the criteria required for board recertification. As LLM technology continues to progress, its prospective applications in surgical education, training, and CME are expected to improve patient outcomes and safety. However, it is important to note that these findings are based on a limited set of multiple-choice questions and may not fully represent the complexity of real-world surgical practice. The exclusion of visual elements and the lack of comparison with human performance limit the generalizability of our results.

Further research is necessary to explore the effects of LLMs on the acquisition and retention of surgical skills as well as on clinical decision-making. Future studies should aim to compare AI performance with that of human surgeons at various levels of expertise, incorporate visual elements in the assessment, and evaluate the models' performance in more diverse and complex scenarios. Additionally, the ethical implications and potential biases of AI in medical education and practice warrant continued scrutiny and investigation. Despite these limitations, our study provides valuable insights into the potential of AI in cardiothoracic surgical education and sets the stage for further exploration of this rapidly evolving field.
